# Advancing Structural Reinforcement in 3D-Printed Concrete: Current Methods, Challenges, and Innovations

**DOI:** 10.3390/ma18020252

**Published:** 2025-01-08

**Authors:** Bo Nan, Youxin Qiao, Junjie Leng, Yikui Bai

**Affiliations:** College of Water Conservancy, Shenyang Agricultural University, Shenyang 110866, China; nanbo@syau.edu.cn (B.N.); 2023240202@stu.syau.edu.cn (Y.Q.); 2023240185@stu.syau.edu.cn (J.L.)

**Keywords:** 3D-printed concrete, steel reinforcement, fiber reinforcement, nano-material reinforcement, interlayer adhesion

## Abstract

With rapid global urbanization and economic development, 3D concrete printing (3DCP) technology has emerged as an innovative construction method, garnering increasing attention and application. Compared to traditional construction techniques, 3DCP not only reduces resource waste and carbon emissions during the building process but also significantly enhances construction efficiency, demonstrating considerable potential in the construction industry. As 3DCP advances from theoretical studies to real-world applications, providing stable and reliable structural reinforcement solutions becomes essential. This paper focuses on examining various methods to improve the performance of 3D-printed concrete (3DPC) structures. The analysis shows that reinforcement using steel bars, in combination with other materials (mainly fibers and nanomaterials), remains a key strategy for structural enhancement. By integrating diverse reinforcement methods, this study proposes an innovative bidirectional steel mesh layout scheme. Additionally, given the unique nature of 3DPC construction, a critical review of various methods for improving interface bonding strength is included. These findings aim to guide the engineering community in selecting suitable reinforcement solutions for 3DPC.

## 1. Introduction

**Figure 1:**
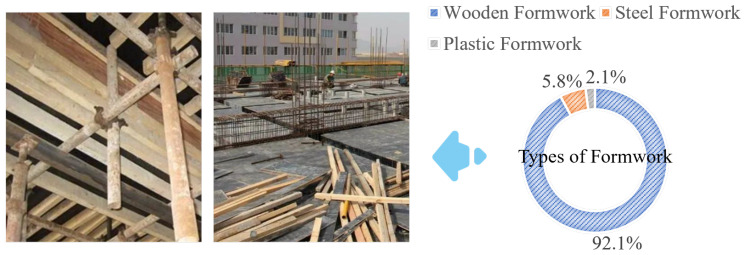
Distribution of different types of frameworks in construction.

The construction industry plays a vital role in driving global urbanization, industrialization, and economic development. However, its growth has been accompanied by significant challenges related to resource consumption, energy use, and environmental impact. According to statistics, the construction industry accounts for 40% of global energy consumption, 40% of solid waste, and 38% of greenhouse gas emissions [1]. The traditional casting method, illustrated in Figure 1, involves assembling large quantities of formwork. Due to its low cost, wooden formwork constitutes over 90% of the formwork used in the Chinese construction industry. The production and processing of wooden formwork require substantial labor, consume significant amounts of energy, and contribute to carbon emissions. Studies have shown that the labor and material costs associated with wooden formwork production account for 25% to 35% of the total cost of concrete structures [2]. The carbon emissions generated by using wooden formwork can reach approximately 14% of the total carbon emissions produced per cubic meter of poured concrete [3], and the resulting formwork waste constitutes about 23% of the total material waste during construction [4]. When considering factors such as electricity and water consumption, as well as transportation during traditional construction methods, it has been found that the carbon emissions from labor amount to 312 tCO_2_e, approximately 4.7 times the carbon emissions produced by construction machinery transportation and operation [5]. The advent of 3D concrete printing (3DCP) technology offers a solution to these issues. With its high degree of informatization and automation, 3DCP reduces the demand for construction labor by 50% to 80%, resulting in a 64.1% decrease in total carbon emissions from construction workers [6]. Furthermore, since 3DCP eliminates the need for formwork, it reduces resource waste associated with formwork disposal [7] and enables the creation of more complex structures [8]. Regarding environmental sustainability, as proposed by W. Zhou, the CSA-ECC mix has a carbon footprint of merely 69% of that of concrete [9].

**Figure 2:**
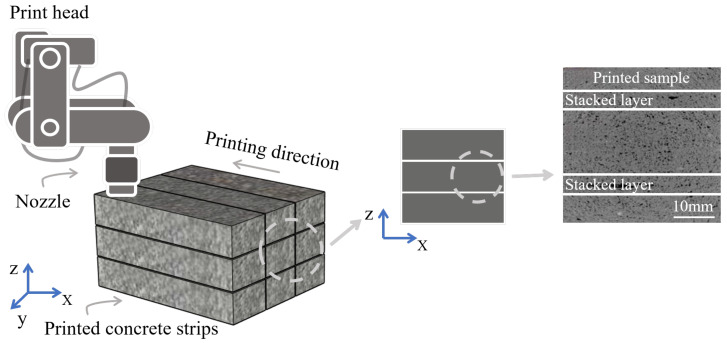
Three-dimensional-printed concrete stacking construction process.

However, the layer-by-layer stacking arrangement inherent to 3D-printed structures, in contrast to the monolithic casting of traditional methods, results in insufficient tensile, shear, and flexural strengths for non-reinforced structures [10]. Furthermore, this construction approach creates a more complex microstructure at the interfaces between layers and strips compared to conventional cast-in-place concrete [11] (see Figure 2), which weakens overall structural integrity and induces anisotropy in the structure [12,13]. Due to issues such as inconsistent layering and weak interlayer bonding, 3DPC may not always achieve the same strength and durability as traditionally cast concrete, limiting its widespread application in large-scale concrete construction. However, it is important to note that the rapid construction capabilities of 3DCP offer significant potential for low-rise, remote housing reconstruction projects [14]. M. Batikha’s study demonstrates that, excluding prefabricated modular concrete, 3DCP can reduce construction duration by approximately 95% [15]. Consequently, to fully leverage this advantage while ensuring that the strength and durability of 3D-printed buildings meet the required standards, research on reinforcing 3DPC structures is essential [16,17]. This paper reviews three primary reinforcement strategies for 3DPC, categorized by the materials used: (1) reinforcement with steel materials; (2) reinforcement with fiber materials; and (3) reinforcement with nanomaterials. The discussion examines the decline in interfacial bonding performance resulting from the unique construction process of 3DPC and provides a comprehensive overview of various methods to enhance bonding performance.

## 2. Material Properties of 3D-Printed Concrete

**Figure 3:**
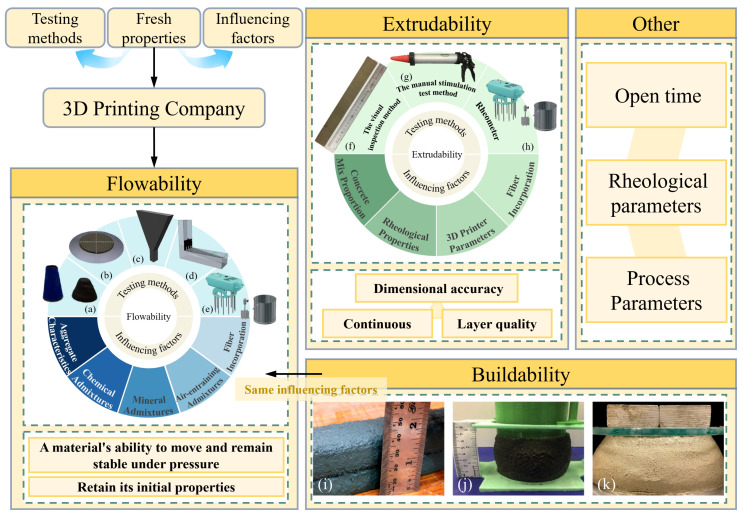
Material parameters and testing methods affecting 3DPC fresh properties: (**a**) slump tests; (**b**) flow table tests; (**c**) V-funnel tests; (**d**) L-box tests; (**e**,**h**) rheometers; (**f**) visual observation; (**g**) specific extrusion tool; (**i**) the layer settlement test; (**j**,**k**) stability tests of cylindrical specimens.

The cementitious material used in 3D printing comprises components such as cementitious binders, aggregates, admixtures, fiber materials, and chemical additives [18,19]. However, its mix proportions differ significantly from those of traditional cast concrete [20,21,22]. The flow characteristics and evaluation methods of 3DPC mixtures are critical, as the fresh state of 3DPC imposes distinct performance requirements compared to cast concrete. This section discusses key material parameters, including flowability, extrudability, buildability, and open time [23]. Furthermore, the fresh properties of 3DPC are influenced by factors such as the printing process and rheological parameters [24], as illustrated in Figure 3.

### 2.1. Flowability

The flowability of 3DPC mortar is a critical parameter that determines whether the mixture is suitable for printing. It refers to the material’s ability to maintain stability under pressure while preserving its initial properties [25]. Flowability is crucial in ensuring that the material can flow smoothly through pipes and other delivery equipment during pumping. Good flowability ensures uniform and continuous movement of the material [26], preventing blockages or segregation. As such, the characteristics of aggregates play a key role in determining the flow properties of the fresh material. Coarse aggregates with large particle sizes can cause blockages in the pipeline, which is why they are rarely used in 3DPC mixtures [27]. Instead, waste materials, such as concrete and brick debris from demolished buildings, are commonly used to achieve similar effects to those of coarse aggregates [28,29]. In addition to these primary factors, the dosage of chemical additives (such as superplasticizers, retarders, and accelerators) [30], air-entraining agents (to improve flowability) [31], mineral admixtures (such as blast furnace slag) [32], and fibers (which can reduce flowability as fiber content increases) [33] also influence the flow properties of the mixture. This makes it essential to measure and control the flowability accurately. Common testing methods include slump tests (Figure 3a), flow table tests (Figure 3b) [34], V-funnel tests (Figure 3c), L-box tests (Figure 3d) [35], and the use of rheometers to measure yield stress and plastic viscosity, which quantify the flowability of the mixture (Figure 3e) [36].

### 2.2. Extrudability

During the printing process, the extrudability of the 3DPC mixture is a crucial parameter [37]. It refers to the ability of the mixture to be continuously extruded through the nozzle while maintaining dimensional accuracy and layer quality [38]. Due to the inherent limitations of the construction method, 3DPC naturally contains pores between printed layers. Ensuring the quality of the extruded layers involves minimizing large voids and fractures, which could compromise the structural integrity [39]. From a rheological perspective, the material should exhibit low viscosity in its liquid state within the pump and nozzle, while the yield stress, which exceeds the self-weight stress after extrusion, allows the material to resist deformation and solidify rapidly [40]. Similar to flowability testing, the yield stress of 3DPC materials can be measured using a rheometer to quantify the extrudability of the material [41]. If the yield stress is too high, the material will be difficult to extrude, preventing the formation of continuous strips. Conversely, if the yield stress is too low, the extruded strips will fail to maintain their shape and will be unable to support subsequent layers of printing. The yield stress of the mixture can be effectively adjusted by modifying the concrete mix design [42,43,44], fiber content [45,46], and printer parameters [47]. In this context, A. Rahul [40] found that the optimal yield stress range for 3DPC materials suitable for printing is 1.5–2.5 kPa, while K. El Cheikh [47] recommended that the diameter of the printing nozzle should be at least 4.25 times the maximum diameter of the solid particles. Simpler methods for evaluating extrudability, such as visual observation (Figure 3f) [48] and extruding a specified length of material using specific tools to check for breakage (Figure 3g) [49], provide convenient and rapid ways to assess the extrudability of the material.

### 2.3. Buildability

The flowability and extrudability of the mixture must meet the printing requirements before it can be applied in actual construction. However, the buildability of the material depends on more than just these factors. Due to the stacking nature of 3DPC, the extruded layers must maintain a uniform geometric shape under the self-weight of the continuously deposited layers [50]. Currently, there is a lack of standardized testing methods and quantifiable indicators for buildability. Conventional assessments are typically conducted through visual observation, using methods such as the layer settlement test (shown in Figure 3i) [40] and stability tests of cylindrical specimens (shown in Figure 3j,k) [51,52].

The performance parameters of 3DPC materials are crucial not only for ensuring the smooth progression of the printing process but also for directly influencing the overall strength and stability of the final structure. Precisely controlling these parameters can minimize structural defects during construction, thereby enhancing the durability and load-bearing capacity of the building. Although research on these properties has made some progress, further exploration of effective enhancement methods is still necessary to improve the performance of 3D-printed building structures.

## 3. Steel Reinforced 3D Printed Concrete

### 3.1. Manual Reinforcement

In traditional cast-in-place construction, the use of rebar cages is commonly applied in large-scale buildings [53]. Reinforced concrete structures are among the most widely used types in the construction industry, which has prompted some 3DPC research to focus on similar reinforcement methods. The selection of steel materials for reinforcement can be categorized into rebar, steel cables, and steel meshes [54,55].

**Figure 4:**
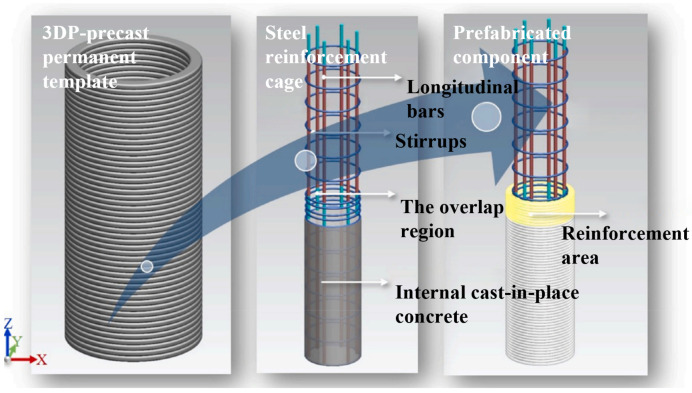
Fabrication process of 3DPC column specimens [59].

A commonly used and simpler construction method involves printing concrete formwork using a contour crafting technique [56], followed by placing a pre-assembled rebar cage inside and pouring concrete to form a composite structure (Figure 4). This approach creates a structure similar to traditional cast-in-place construction, enabling effective cooperation between the rebar and concrete in load-bearing. Another method involves pre-printing rebar ducts during the printing process and, after the concrete hardens, post-tensioning the prestressed tendons [57,58] to enhance the structure.

**Figure 5:**
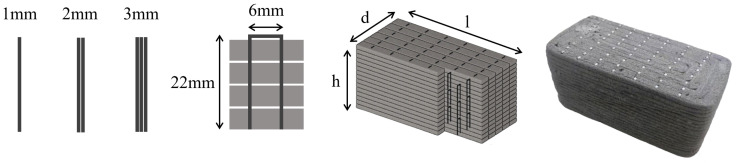
U-shaped staple reinforcement.

**Figure 6:**
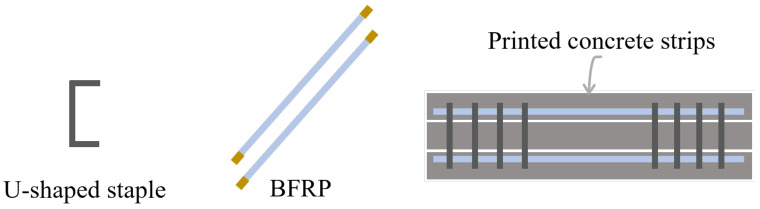
BFRP-reinforced concrete specimen.

Unlike traditional rebar cage forms, L. Wang [60] proposed a method that incorporates U-shaped nails as vertical reinforcement, placed synchronously with the 3D printing process to improve interlayer bonding (Figure 5). The dimensions of a single U-shaped nail are 6 mm in width, 22 mm in length, and 1 mm in thickness. By combining multiple U-shaped nails of different thicknesses, the optimal thickness was found to be 2 mm to 2.5 mm. When loaded along the Y-direction (subsequent loading direction coordinates should refer to Figure 2), the bridging effect of the U-shaped nails led to a 37.8% to 61.8% increase in interlayer tensile bonding strength, while the interlayer shear bonding strength increased by up to 120% due to the pinning effect of the nails. Using a similar approach, C. Gao [61] employed U-shaped nails as stirrups to secure BFRP (Basalt Fiber Reinforced Polymer) bars (Figure 6), loaded along the Z-direction, achieving a 20% to 113% increase in the bearing capacity of printed beams as the reinforcement ratio increased from 0.438% to 1.381%.

**Figure 7:**
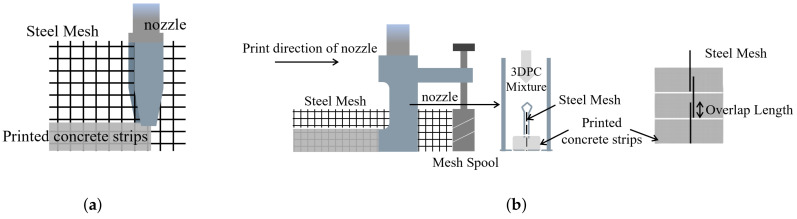
Two schemes of longitudinal reinforcement with wire mesh: (**a**) a fork-shaped nozzle; (**b**) The improved fixed-height steel mesh synchronous reinforcement device.

In addition to using rebar for structural reinforcement, steel wire mesh can also form a reinforcement structure similar to a rebar cage. Huashang Tenda Company [62] developed a fork-shaped nozzle (Figure 7a) that extrudes concrete strips directly on both sides of pre-placed steel wire mesh. This method of integrating steel wire mesh into the printing process offers high flexibility but is limited by the height of the fork-shaped nozzle, which restricts the construction of taller structures. To address this limitation, T. Marchment [63] proposed an improved design (Figure 7b), using a fixed-height nozzle while stacking 26 mm high, 6 mm × 6 mm steel wire mesh grids.

**Figure 8:**
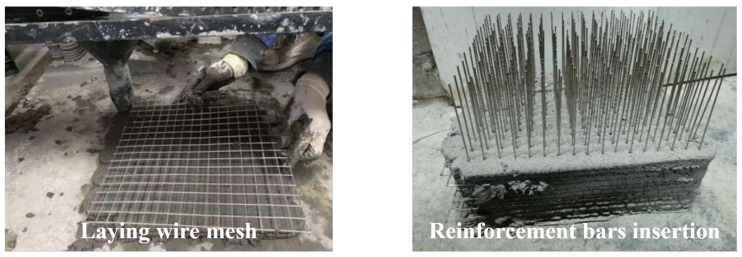
Scheme of transversely placed wire mesh with longitudinal dowel bars.

In contrast to placing mesh reinforcement vertically between layers, A. K. Akhnoukh’s team [64] suggested placing the mesh horizontally at the interlayer defects, applying this method in real engineering projects. While this approach significantly enhances the mechanical properties of 3DPC, its effectiveness in vertical reinforcement remains limited, necessitating further research. In response, T. Zhang [54] proposed a method of placing steel wire mesh between layers with vertical dowels inserted in the stacking direction for structural enhancement (Figure 8). This dowel concept is inspired by T. Marchment’s [65] approach of inserting rods vertically after printing. This reinforcement method improved the compressive strength of 3D-printed concrete specimens in the Z-direction by 25% and the tensile strength by 500%, prompting T. Zhang to propose a prefabricated negative pressure medical cabin design [66]. Unlike traditional casting construction, where contour printing is used, the approach of incorporating steel wire mesh requires consideration of its impact on the interlayer bonding behavior of the concrete. Current research on this aspect remains limited [67,68].

**Figure 9:**
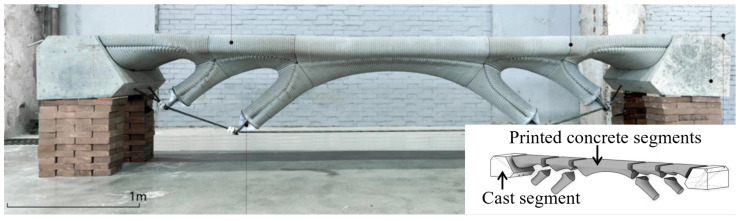
Steel cable-reinforced 3DPC bridge.

All the aforementioned methods aim to create a “rebar-like cage” structure for structural reinforcement. A more innovative method involving steel cables was proposed by J. Xiao’s team [69]. Similarly, L. Yu’s team [55] used tensioned steel cables to support the lower structure (Figure 9), enabling 3D-printed concrete components to achieve high performance without additional reinforcement, making them particularly suitable for bridge structures or roof slabs.

### 3.2. Automated Reinforcement

**Figure 10:**
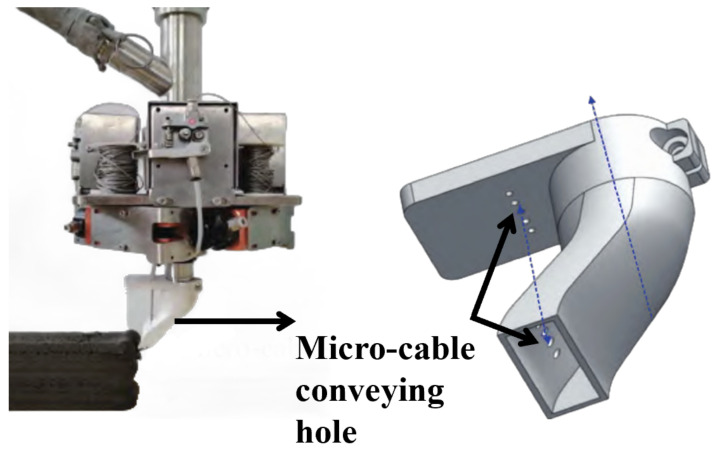
Synchronous micro-reinforcement device for 3DPC.

Existing reinforcement methods predominantly rely on manual rebar placement, resulting in low automation levels. To further reduce labor usage and carbon emissions, various approaches with higher degrees of automation have emerged. These approaches can be categorized into two types: (1) non-synchronous automated reinforcement using robotic arms and automated equipment and (2) synchronous reinforcement during the 3D printing of concrete layers. M. Alabbasi [70] utilized parametric modeling, topology optimization, finite element analysis, and robotic 3D printing tools to achieve large-scale customization of typical standalone houses in Saudi Arabia. Their study proposed an innovative design-to-manufacture framework that integrates 3D concrete printing with robotic manufacturing, enabling a fully automated construction process. Compared to construction robots, adding reinforcement materials synchronously during 3D printing offers a more cost-effective construction method. Z. Li [71] developed a system that embeds rebar during the printing process by installing a device on the material hopper to extrude steel wires. Similarly, the team at Hebei University of Technology [72,73] proposed a multi-rebar synchronous reinforcement system for 3DPC, utilizing a print nozzle equipped with micro-rebar channels (Figure 10) to enhance operational convenience. Four-point bending tests on specimens demonstrated that the inclusion of rebar increased the ultimate load-bearing capacity by 220% under bending loads in the Z-direction.

From the current development of 3DPC, placing rebar cages inside the structure after contour printing and subsequently pouring concrete remains the most practical and feasible reinforcement method. This approach leverages the mold-free construction advantage of 3DPC while maintaining consistency with traditional reinforced concrete structures, allowing the use of existing structural design codes. However, the automation level of this method is still low, as manual tying of rebar cages is required. Amid the global push for intelligent construction, the construction industry is undergoing unprecedented transformation [74]. Integrating digital technologies such as Building Information Modeling (BIM) [75] to develop comprehensive, intelligent, and automated construction systems represents the future direction and trend for advancing 3DPC technology.

## 4. Fiber and Nanomaterial-Reinforced 3DPC

### 4.1. Types of Fibers and Their Applications in 3DPC

The use of fibers to control crack propagation in concrete has been widely implemented in traditional reinforced concrete structures. For 3DPC structures, fiber reinforcement methods can be broadly categorized into two types: (1) weaving continuous long fiber strands into structural elements as a substitute for steel mesh and (2) incorporating short fibers uniformly into the printing material mixture.

Common fiber types used in 3DPC reinforcement include polymer fibers, basalt fibers, steel fibers, glass fibers, and carbon fibers [76,77,78,79,80]. The incorporation of fibers must balance the requirements of flowability, extrudability, and buildability in 3DPC, necessitating the determination of optimal mixing ratios. B. Li [81] added polyvinyl alcohol fibers (PVAF) to iron tailings concrete, showing that under a constant water-to-binder ratio, the compressive strength of 3DPC decreased as PVAF content increased, while flexural strength initially improved but declined with further fiber addition. C. Zhang [82] explored the effects of polyvinyl alcohol fibers (PVAF), polypropylene fibers (PPF), and sisal fibers (SSF) on the rheological, printable, and mechanical properties of 3DPC. Their findings revealed that a 0.5% (volume fraction; the same applies below) addition of PVAF significantly increased static yield stress by 308% compared to the control group, though higher fiber content led to extrusion failure. PVAF also enhanced dynamic shear stress and plastic viscosity, while PPF and SSF had minimal effects on these parameters. Triaxial compression tests indicated that compressive strength declined with increasing PVAF or SSF content, whereas PPF demonstrated the most pronounced strengthening effect, with an optimal content range of 0.1% to 0.3%. Due to the rough surface of sisal fibers, their interaction with the concrete matrix was stronger, resulting in a flexural strength increase exceeding 30%.

In addition to the aforementioned flexible fibers, rigid fibers have also attracted significant attention from many scholars. B. Panda [83] found that incorporating 1% short glass fibers (GF) of 3 mm length effectively enhanced the tensile and flexural strength of 3DPC specimens, though it slightly reduced compressive strength. Carbon fiber (CF), a high-performance material known for its high strength, rigidity, lightweight nature, and resistance to corrosion and heat, maintains stable performance under high loads and harsh environments. Its exceptional fatigue resistance and design flexibility have led to widespread applications in aerospace, automotive, wind energy, construction engineering, and medical devices. M. Hambach [84] investigated CF incorporation and found that its spatial distribution through the nozzle achieved similar enhancement effects to GF at a 1% addition level. However, due to its superior toughness, CF had a more pronounced impact on flexural strength than GF. Y. Zhao and X. Wu [85,86] observed that increasing basalt fiber (BF) content from 0.1% to 0.5% significantly improved rheological properties and enhanced 28-day flexural strength by over 18%. Compressive strength showed a positive correlation with BF content at 7 days but initially increased and then declined at 28 days due to uneven fiber distribution, reduced workability, and increased internal porosity. Research on steel fibers (SF) referenced the works of J. Xia [87] and J. Hu [88], who recommended an optimal fiber content of 2% to 3%. Their results demonstrated that this range led to a compressive strength increase of over 35% and tensile strength improvements exceeding 130%. Hu Jinhan also conducted flexural and splitting tensile tests, showing significant enhancements in flexural performance and interlayer splitting strength, with increases of 75.63% and 26.39%, respectively.

As shown in Table 1, different reinforcement materials exhibit varying effects on strength enhancement. Flexible fibers generally have a minimal or even negative impact on compressive strength, while rigid fibers, due to their high strength and rigidity, significantly improve the compressive strength of the specimens. Similarly, fibers enhance the flexural performance of 3DPC to varying degrees, as they act as bridges between concrete layers, effectively inhibiting crack formation. However, excessive fiber content may have adverse effects.

An alternative reinforcement method involves replacing steel mesh with a structure woven from continuous long fibers to enhance the structural integrity. A. Ramesh [89] conducted research similar to the proposal by Huashang Tengda Company, utilizing alkali-resistant glass fiber mesh in place of steel mesh for reinforcement. The study demonstrated that fiber textile reinforcement improved the load-bearing capacity by approximately 60%. Additionally, under bending stress, the textile-reinforced samples exhibited substantial deflections and delayed crack formation, showcasing their superior energy dissipation and structural toughness. Although further in-depth research is needed to fully explore the application of woven fibers in large concrete buildings, their considerable potential has already attracted significant attention. Notably, woven fibers can drastically reduce the weight of the structure, which is essential for lightweight design. Lightweight design not only lowers construction costs but also enhances construction efficiency and reduces environmental impact. Furthermore, the high strength and exceptional flexibility of woven fibers provide increased tensile strength and toughness, thereby improving the overall stability and seismic performance of the building. These properties present new opportunities for designing future large concrete structures, promoting technological advancement and innovation in the construction industry.

### 4.2. Applications of Nanomaterials in 3DPC

**Figure 11:**
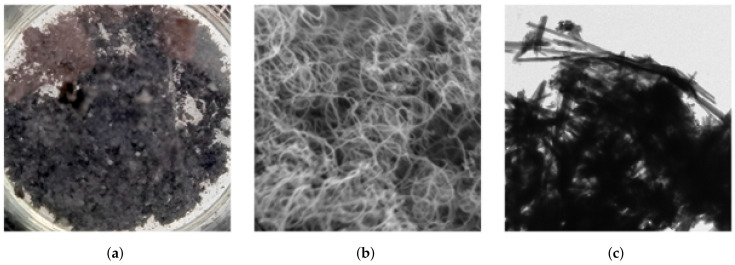
(**a**) CNTs; (**b**) MWCNTs; (**c**) HNTs.

In current concrete material research, alongside the use of traditional fibers to control crack propagation at the macroscopic scale, there is a growing emphasis on utilizing nanofibers to address microcracks within the microstructure [90]. Figure 11 illustrates several common types of nanomaterials.

Existing research has demonstrated that the incorporation of carbon nanotubes (CNTs) can significantly improve the quality and mechanical properties of concrete, enhancing its performance in terms of strength, toughness, and durability [91]. M. Ali [92] investigated the crack-bridging role of CNTs in the cement matrix, and their experiments confirmed that the combination of CNTs with superplasticizers significantly improved the printability, constructability, and rheological properties of the material. The study identified the optimal mix ratio and its effects on print quality and structural stability, with the addition of 0.2% CNTs showing the most significant improvement. Microscopic analysis further confirmed that CNTs were uniformly dispersed in the cement matrix, enhancing the interaction between particles and significantly increasing both compressive and flexural strength. In studies on the effects of multi-walled carbon nanotubes (MWCNTs) on 3D-printed concrete [93,94], A. Dulaj explored the effects of different MWCNT concentrations on the material’s porosity, mechanical properties, and electrical resistivity. The results showed that MWCNT distribution and concentration significantly influenced these properties. Notably, the 0.5% MWCNTs composition exhibited the best performance in both fresh and hardened states, demonstrating excellent self-sensing capabilities and revealing its potential for improving material strength and monitoring structural health. Similarly, M. Razzaghian and N. Ranjbar [95,96] evaluated the fresh-state properties, hardening performance, and microstructural characteristics of 3D-printed concrete incorporating halloysite nanotubes (HNTs). N. Ranjbar examined the use of HNTs and their calcined products in 3D-printed geopolymer mortars, finding that the addition of 1–2 wt% HNTs, due to their unique nanotube structure that absorbs moisture and increases friction between particles, significantly improved the rheological properties and buildability of the mortar. Calcined HNTs, with their high reactivity, also accelerated the setting time of the geopolymer. M. Razzaghian found that incorporating 3% HNTs significantly improved the mechanical properties of 3DPC mortar by reducing internal porosity, leading to more than a 39% increase in both compressive and flexural strength in the Z-direction and a 19% improvement in interlayer bonding strength when tested along the Y-direction. These studies not only provide new insights for optimizing 3D-printed cement materials but also lay the foundation for the development of future smart construction materials.

## 5. Methods to Improve Interlayer Adhesion in 3DPC

The layer-by-layer construction method of 3D-printed concrete can lead to issues such as weak interlayer bonding [97] and shrinkage cracking [98,99], which pose significant challenges to its structural application [100]. Interlayer bonding refers to the adhesion between freshly extruded concrete and the previously laid, solidified layers during the printing process. This bonding is influenced by factors such as the humidity of the printed layers [101], the interval between printing layers [102], and the properties of the concrete mix [103]. To address these challenges, many researchers have proposed innovative solutions for local reinforcement between layers, achieving notable advancements and applications in this area [104,105].

**Figure 12:**
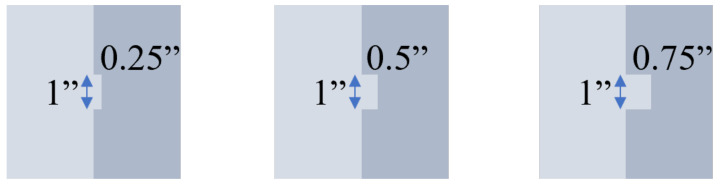
The size of the interlocking.

Common methods for enhancing the interlayer interface can be categorized based on their mechanisms of action: (1) physical structure reinforcement and (2) the addition of supplementary materials. B. Zareiyan and B. Khoshnevis [106] proposed a physical interlocking structure (IS) between the upper and lower layers of concrete strips (Figure 12) to investigate the strength development associated with different interlocking depths at 28 days. The results demonstrated that varying interlocking depths positively influenced structural performance, with strength improving over time. The optimal enhancement of compressive strength and interlayer bonding was achieved at an interlocking depth of 0.75 inches, as shown in Table 2. However, implementing such a structure in practical construction is challenging. B. Zareiyan and B. Khoshnevis suggested that a similar effect could be achieved by using specialized tools to create a serrated surface on the printing layer.

**Figure 13:**
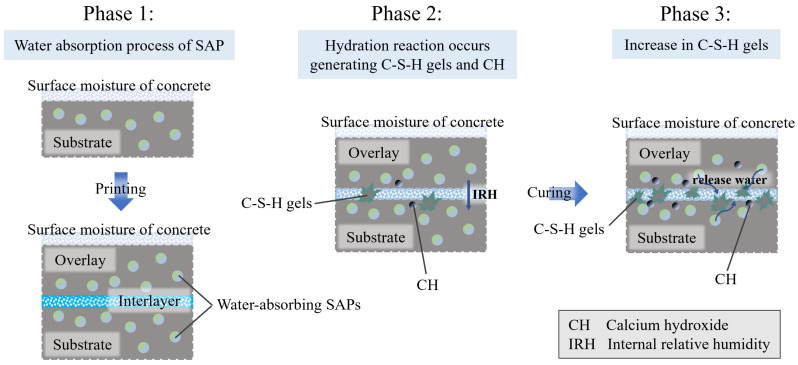
Mechanism of action of SAP (The surface moisture of concrete and the color intensity of the interlayer indicate that a darker color represents a higher moisture content).

**Figure 14:**
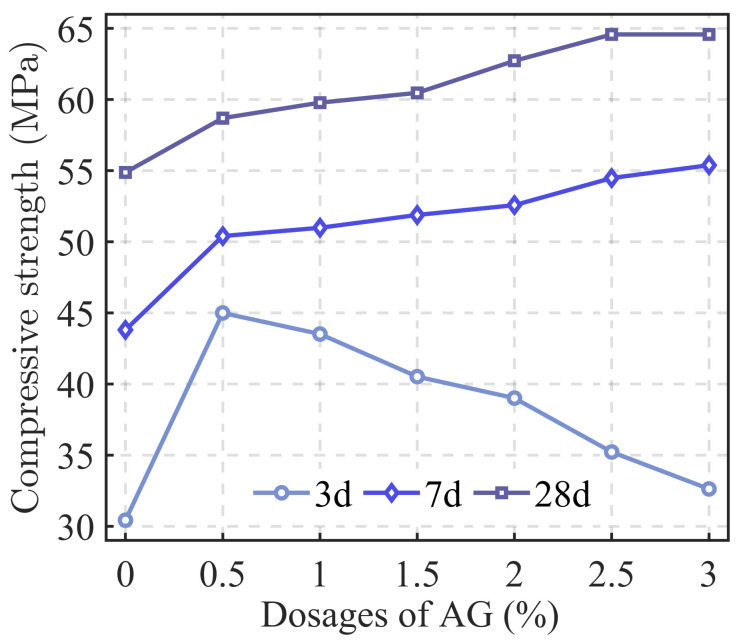
Compressive strength of specimens with different dosages of AG.

G. Moelich [107] investigated the impact of different types of superabsorbent polymers (SAPs) on the interlayer bonding performance of 3DPC. The water-retaining SAP exhibited the most significant enhancement, and a predictive model for surface moisture-interlayer bond strength was developed. Building on this, Q. Luo [108] studied the effects of various SAPs on the early mechanical and interlayer bonding properties of 3DPC. The results showed that the early compressive strength of the specimens significantly improved with the addition of water-retaining SAP, and this improvement positively correlated with curing time, although the growth rate slowed over time. Regarding interlayer bonding, the research team proposed using layered casting and tensile tests to efficiently and effectively evaluate interlayer bonding performance in 3DPC. The results showed growth characteristics similar to early compressive strength, with the SAP enhancement mechanism illustrated in Figure 13. T. Pan [109] incorporated 8% nanoclay (Nc) into 3D-printed concrete mortars containing either polycarboxylate superplasticizer (PCE) or their self-synthesized slow-setting carboxylate superplasticizer (TS) and tested the interlayer bond strength for both groups. The study indicated that both superplasticizers significantly improved the bonding strength of weak interlayer zones. The bonding strength gradually increased as curing time extended from 3 to 28 days, with TS showing better performance. After 3 days of curing, the interlayer bonding strength of the TS group increased by 24.12% compared to the PCE group, and although the enhancement slowed at 28 days, it still showed a 2.13% improvement over PCE. H. Yao’s team [110] introduced attapulgite (AG) into 3D-printed concrete mortar and measured the compressive strength of specimens at different curing times. The results showed that, except at 3 days, the compressive strength of the specimens increased with the amount of AG added at 7 and 28 days. The phenomenon observed at 3 days was explained by the addition of a certain amount of superplasticizer to ensure the required flowability for early-stage cement mortar extrusion (Figure 14). Interlayer bonding tests revealed that the small particle size of AG filled the interface pores, making the connection smoother. This caused AG to negatively impact interlayer bonding performance, with bond strength negatively correlated with the amount of AG added.

Y. Tao [111] explored the application of chemical reaction coatings to enhance the interlayer bonding performance of 3D-printed concrete. In this study, a dual-component mixture of calcium sulfoaluminate (CSA) cement and Portland cement was prepared, and CSA cement paste was applied at the interface. The effectiveness of the chemical reaction coating was evaluated through strength tests. Although the enhancement was not as significant as SAP, the CSA cement coating rapidly underwent hydration upon activation, hardening the material at the interface and filling the voids, forming a strong bond. This approach demonstrated great potential for the automated repair of concrete structures.

**Figure 15:**
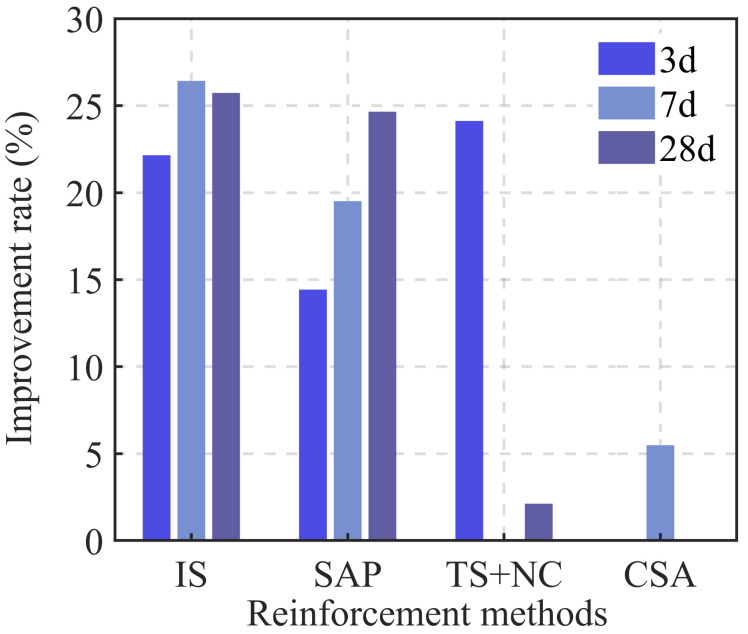
Interlayer adhesion improvement rate compared to the control group with the same curing age.

As shown in Table 2 and Figure 15, all materials, except for AG, which causes a reduction in interlayer adhesion, exhibit some degree of enhancement in interlayer bonding. Among these, the interlocking structure and SAP show the most significant improvements at various curing ages. Notably, SAP has a particularly pronounced effect on early compressive strength, contributing substantially to the buildability of 3DPC.

## 6. Discussion and Future Research Directions

**Figure 16:**
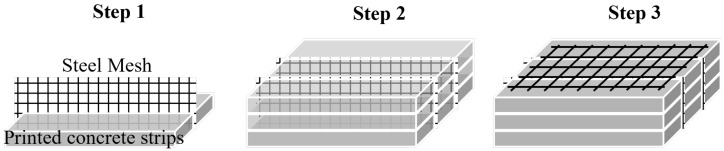
Improved steel mesh reinforcement.

As shown in Figure 16, by integrating diverse reinforcement methods, this study proposes an innovative bidirectional steel mesh layout scheme. By combining the previously mentioned vertical placement method with the horizontal laying technique, reinforcement is achieved by printing multiple layers of concrete strips at the same height on both sides of a fixed-height steel mesh. After completing the printing of the entire bottom surface, the steel mesh is placed horizontally to cover the surface, and the printing operation is repeated, ultimately completing the structural reinforcement.

Due to the requirement for fork-shaped nozzles matching the height of the steel mesh and the limitation on infinitely increasing nozzle height, this scheme adopts the method proposed by Section 3, which involves placing vertically oriented steel mesh of fixed height. By overlapping sections between upper and lower mesh layers, a structure similar to a continuous reinforced mesh is achieved. Additionally, transverse steel mesh is laid in alternating layers, where the grid of the transverse mesh provides a stirrup-like effect on the vertical mesh, limiting its lateral displacement to a certain extent.

**Figure 17:**
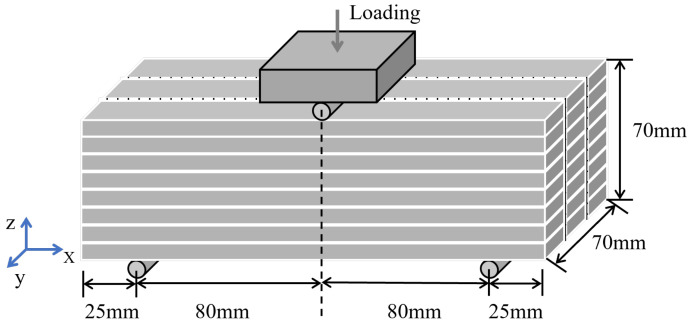
Loading schematic diagram.

A preliminary specimen, measuring 70 × 70 × 210 mm (Figure 17), was fabricated and tested under loading along the Z-direction. The steel mesh utilized featured a 6 mm grid spacing and 1 mm diameter wires.The printing mixture utilizes 42.5 fast-setting, early-strength sulfoaluminate cement, combined with the ingredients listed in Table 3. The reinforcement material consists of 304 stainless steel mesh.

**Figure 18:**
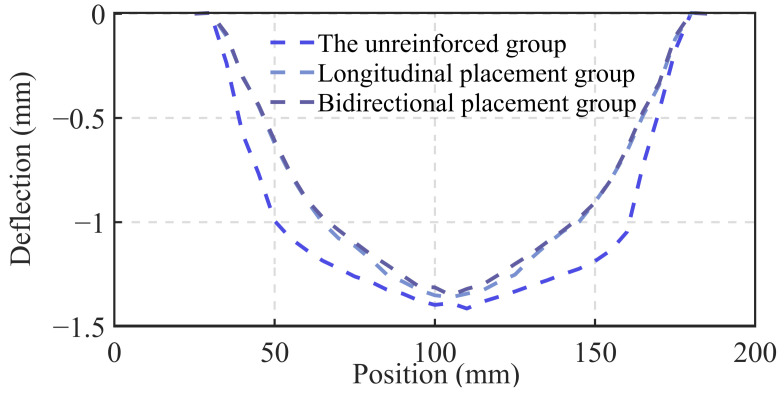
Comparison of deflection at the bottom surface of samples with different reinforcement methods.

**Figure 19:**
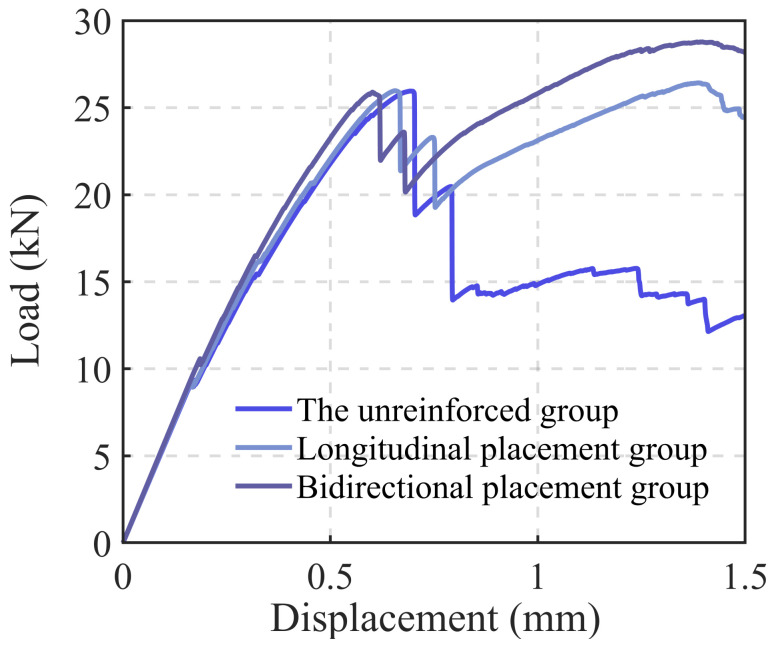
Displacement-load curve of bending specimens in the Z-direction.

Through a comparative analysis of the unreinforced group and the groups with unidirectional and bidirectional steel mesh reinforcement, as shown in Figure 18, the maximum displacement of the reinforced samples is 3% to 5% lower than that of the unreinforced samples. Additionally, the overall displacement curve is significantly flatter, indicating superior structural toughness. The bidirectional reinforcement samples exhibit slightly lower maximum displacement compared to the unidirectional reinforcement samples. As shown in Figure 19, when the load reaches approximately 25.8 kN, all three sample groups display two distinct drops. This behavior can be attributed to the specimen cutting principle, where more complete concrete strips are retained in the middle layers, causing the upper and lower strips to become thinner. After the lower concrete layer loses its load-bearing capacity, the steel mesh assumes the load, leading to the emergence of an upward segment. The peak load of the bidirectional reinforcement group reaches 28.78 kN, representing a 10.88% increase compared to the unreinforced group and an 8.91% increase compared to the unidirectional reinforcement group. The descending segment reveals that all reinforced groups exhibit good structural toughness, with the bidirectional reinforcement group showing the best performance.

Current research preliminarily demonstrates the feasibility of the bidirectional steel mesh placement method. Future studies will further investigate factors influencing bidirectional reinforcement, such as wire diameter and mesh spacing. In-depth research into the bond behavior between steel mesh and concrete will be conducted to identify optimal reinforcement parameters, with the goal of providing theoretical guidance for low-rise building reconstruction in remote areas.

## 7. Conclusions

With the rapid advancement of 3D-printing technology in concrete construction, its application in large-scale buildings presents unique challenges. To fully harness the potential of 3DPC technology, extensive research into structural reinforcement is crucial for enhancing the performance and stability of concrete structures. This paper reviews current reinforcement methods for 3D-printed concrete and evaluates the effectiveness of each approach. Reinforcement with steel bars, supplemented by fibers and nanomaterials, remains a key method for structural enhancement. Our study resulted in the following conclusions:

1. The 3DPC reinforcement method that forms a “reinforced cage-like” structure differs from traditional casting methods. During the printing process, the use of wire mesh reinforcement requires consideration of its effect on the interlayer bonding behavior of the concrete. Currently, research primarily focuses on the bond between concrete layers, with limited attention given to the influence of wire mesh. Further exploration in this area could provide valuable insights.
2. Research on the synergistic effects of reinforcement methods, such as wire mesh, fiber materials, and nanomaterials, remains underdeveloped. The future of 3D-printed concrete construction should prioritize the interaction between different reinforcement materials. By conducting in-depth research on wire mesh, fiber materials, and nanomaterials, more effective combinations can be identified to guide practical engineering applications.
3. Section 6 introduces a method that effectively combines the advantages of both approaches, while reducing the number of horizontally placed steel meshes, thereby minimizing the weak layers between structural layers. Future research will further investigate the feasibility of the bidirectional steel mesh placement method.

With the maturation of technology and the expansion of its applications, 3D-printed concrete will play an increasingly important role in the future of the construction industry, becoming a powerful catalyst for innovative design and sustainable building practices.

## Figures and Tables

**Table 1:** Reinforcement effects of various fiber materials.

Author	Materials	Improvement Rate (%) ^1^			Optimal Dosage (%)
		Compressive Strength ^2^	Tensile Strength ^2^	Flexural Strength ^2^	
B. Li [81]	PVAF	−0.81	/	6.72	0.25
C. Zhang [82]	PVAF	−5.31	/	2.7	0.1
	PPF	23.30	/	2.7	
	SSF	−3.24	/	30.63	
B. Panda [83]	GF	−12.80	78.36	43.41	1
M. Hambach [84]	CF	−25.28	/	174.53	1
Y. Zhao and X. Wu [85,86]	BF	26.04 (7 d)	/	18.29 (7 d)	0.3
		28.22 (28 d)		25.4 (28 d)	
J. Xia [87]	SF	65.17	131.25	/	2
J. Hu [88]	SF	39.21	149.25	75.63	2.4

^1^ All improvement rates are relative to the control group at the same curing age. ^2^ All testing load directions were in the Z-direction.

**Table 2:** Enhanced effects of various studies.

Author	Reinforcement Methods	Compressive Strength ^2Z^		Interlayer Adhesion ^2Y^	
		Curing Age	Improvement Rate (%) ^1^	Curing Age	Improvement Rate (%) ^1^
B. Zareiyan [106]	Interlocking structure	3 d	17.37	3 d	22.15
		7 d	19.62	7 d	26.42
		28 d	13.09	28 d	25.73
G. Moelich [107]	SAP	/		56 d	10.00
Q. Luo [108]	SAP	15 min	282.17	3 d	14.34
		30 min	174.61	7 d	19.51
		60 min	92.75	28 d	24.65
T. Pan [109]	TS + Nc	/		3 d	24.12
		/		28 d	2.13
H. Yao [110]	AG	3 d	7.23	3 d	−58.48
		7 d	26.43	7 d	−54.43
		28 d	17.67	28 d	−51.14
Y. Tao [111]	CSA	/		7 d	5.49

^1^ All improvement rates are relative to the control group at the same curing age. ^2^ The superscripts Y and Z represent the loading directions of the test specimens.

**Table 3:** Three-dimensional-printed concrete mix proportion.

Cement	Mineral Powder	Silica Fume	Coarse Sand	Fine Sand	Water Reducer	Retarder	PVA Fiber
1	0.8	0.2	0.5	0.1	0.04	0.03	1.2%

## Data Availability

The data presented in this study are available on request from the corresponding author due to privacy.

## Abbreviations

The following abbreviations are used in this manuscript:

3DPC	3D-Printed Concrete
3DCP	3D Concrete Printing
BIM	Building Information Modeling
PVAF	Polyvinyl alcohol fibers
PPF	Polypropylene fibers
SSF	Sisal fibers
GF	Glass fibers
CF	Carbon fiber
BF	Basalt fiber
SF	Steel fibers
CNTs	Carbon nanotubes
MWCNTs	Multi-walled carbon nanotubes
HNTs	Halloysite nanotubes
IS	Interlocking structure
SAPs	Superabsorbent polymers
Nc	Nanoclay
TS	Their self-synthesized slow-setting carboxylate superplasticizer
AG	Attapulgite
CSA	Calcium sulfoaluminate
